# Time inhomogeneous quantum dynamical maps

**DOI:** 10.1038/s41598-022-25694-1

**Published:** 2022-12-08

**Authors:** Dariusz Chruściński

**Affiliations:** Institute of Physics, Faculty of Physics, Astronomy and Informatics, Nicolaus Copernicus University, Grudziadzka 5/7, 87-100 Toruń, Poland

**Keywords:** Quantum information, Quantum mechanics

## Abstract

We discuss a wide class of time inhomogeneous quantum evolution which is represented by two-parameter family of completely positive trace-preserving maps. These dynamical maps are constructed as infinite series of jump processes. It is shown that such dynamical maps satisfy time inhomogeneous memory kernel master equation which provides a generalization of the master equation involving the standard convolution. Time-local (time convolution-less) approach is discussed as well. Finally, the comparative analysis of traditional time homogeneous versus time inhomogeneous scenario is provided.

## Introduction

The dynamics of an open quantum system^1,2^ is usually represented by the dynamical map $\{\Lambda _{t,t_0}\}_{t \ge t_0}$, i.e. a family of completely positive trace-preserving maps $\Lambda _{t,t_0} : {\mathcal {B}}({\mathcal {H}}) \rightarrow {\mathcal {B}}({\mathcal {H}})$^3,4^ (${\mathcal {B}}({\mathcal {H}})$ stands for the vector space of bounded linear operators acting on the system’s Hilbert space ${\mathcal {H}}$). In this paper we consider only finite dimensional scenario and hence ${\mathcal {B}}({\mathcal {H}})$ contains all linear operators. The map $\Lambda _{t,t_0}$ transforms any initial system’s state represented by a density operator $\rho _{0}$ at an initial time $t_0$ into a state at the current time *t*, i.e. $\rho _t = \Lambda _{t,t_0}(\rho _{0})$. Dynamical maps $\{\Lambda _{t,t_0}\}_{t \ge t_0}$ provide the powerful generalization of the standard Schrödinger unitary evolution $U_{t,t_0} \rho _{0} U_{t,t_0}^\dagger$, where $U_{t,t_0}$ is a family of unitary operators acting on ${\mathcal {H}}$. A dynamical map is usually realized as a reduced evolution^1^

1.1 $$\begin{aligned} \Lambda _{t,t_0}(\rho _0) = \textrm{Tr}_E\left( {\mathbb {U}}_{t,t_0} \rho _0 \otimes \rho _E {\mathbb {U}}_{t,t_0} \right) , \end{aligned}$$

where ${\mathbb {U}}_{t,t_0}$ is a unitary operator acting on ${\mathcal {H}} \otimes {\mathcal {H}}_E$, $\rho _E$ is a fixed state of the environment (living in ${\mathcal {H}}_E$), and $\textrm{Tr}_E$ denotes a partial trace (over the environmental degrees of freedom). The unitary ${\mathbb {U}}_{t,t_0}$ is governed by the total (in general time-dependent) ‘system + environment’ Hamiltonian ${\mathbb {H}}_t$. Now, if ${\mathbb {H}}_t={\mathbb {H}}$ does not depend on time the reduced evolution (1.1) is time homogeneous (or translationally invariant), i.e. $\Lambda _{t,t_0} = \Lambda _{t-t_0}$ (or equivalently $\Lambda _{t+\tau ,t_0+\tau } = \Lambda _{t,t_0}$ for any $\tau$). In this case one usually fixes $t_0=0$ and simply considers one-parameter family of maps $\{\Lambda _t\}_{t \ge 0}$. Such scenario is usually considered by majority of authors. The most prominent example of time homogeneous dynamical maps is the celebrated Markovian semigroup $\Lambda _{t} = e^{{\mathcal {L}} t}$, where ${\mathcal {L}}$ denotes the Gorini–Kossakowski–Lindblad–Sudarshan (GKLS) generator^5,6^ (cf. also the detailed exposition in^7^ and^8^ for a brief history)

1.2 $$\begin{aligned} {\mathcal {L}}(\rho ) = - i[H,\rho ] + \sum _k \gamma _k \left( L_k \rho L_k^\dagger - \frac{1}{2} \{ L_k^\dagger L_k,\rho \} \right) , \end{aligned}$$

with the (effective) system’s Hamiltonian *H*, noise operators $L_k$, and non-negative transition rates $\gamma _k$. It is well known, however, that semigroup evolution usually requires a series of additional assumptions and approximations like e.g. weak system-environment interaction and separation of natural time scales of the system and environment. Departure from a semigroup scenario calls for more refined approach which attracts a lot of attention in recent years and is intimately connected with quantum non-Markovian memory effects (cf. recent reviews^9–17^). To go beyond dynamical semigroup keeping translational invariance one replaces time independent GKLS generator ${\mathcal {L}}$ by a memory kernel $\{{\mathcal {K}}_t\}_{t\ge 0}$ and considers the following dynamical equation

1.3 $$\begin{aligned} \partial _t \Lambda _t = \int _0^t {\mathcal {K}}_{t-\tau } \circ \Lambda _\tau d\tau = {\mathcal {K}}_t *\Lambda _t \ , \ \ \ \Lambda _{t=0} = \textrm{id} , \end{aligned}$$

where $A \circ B$ denotes composition of two maps. Equation (1.3) is often referred as Nakajima–Zwanzig master equation^18,19^. The very structure of the convolution ${\mathcal {K}}_t *\Lambda _t$ does guarantee translational invariance. However, the property of complete positivity of $\Lambda _t$ is notoriously difficult as already observed in^20–22^. Time non-local master equation (1.3) were intensively studied by several authors^23–35^. Since the master equation (1.3) involving the convolution is technically quite involved one usually tries to describe the dynamics in terms of convolution-less time-local approach involving a time dependent generator $\{{\mathcal {L}}_t\}_{t \ge 0}$ (cf. the recent comparative analysis^36^). Time-local generator ${\mathcal {L}}_t$ plays a key role in characterizing the property of CP-divisibility which is essential in the analysis of Markovianity. Note, however, that the corresponding propagator $\Lambda _{t,s} = \Lambda _t \circ \Lambda _s^{-1}$ is no longer time homogeneous unless ${\mathcal {L}}_t$ is time independent.

In this paper we go beyond time homogeneous case and consider the following generalization of (1.3)

1.4 $$\begin{aligned} \partial _t \Lambda _{t,t_0} = \int _{t_0}^t {\mathcal {K}}_{t,\tau } \circ \Lambda _{\tau ,t_0} d\tau , \ \ \ \Lambda _{t_0,t_0} = \textrm{id} , \end{aligned}$$

which reduces to (1.3) if ${\mathcal {K}}_{t,\tau } = {\mathcal {K}}_{t-\tau }$. Equation (1.4) may be, therefore, considered as a time inhomogeneous Nakajima-Zwanzig master equation. Such description is essential whenever the ‘system + environment’ Hamiltonian ${\mathbb {H}}_t$ does depend on time. Note, that formally if ${\mathcal {K}}_{t,\tau } = {\mathcal {L}}_t \delta (t-\tau )$, then (1.4) reduces to time-local but inhomogeneous master equation

1.5 $$\begin{aligned} \partial _t \Lambda _{t,t_0} = {\mathcal {L}}_t \circ \Lambda _{t,t_0} , \ \ \ \Lambda _{t_0,t_0} = \textrm{id} , \end{aligned}$$

and the corresponding solution $\Lambda _{t,t_0}$ is CPTP for all *t* and $t_0$ with $t>t_0$ if and only if ${\mathcal {L}}_t$ is of GKLS form for all $t \in {\mathbb {R}}$^1,2,7^. This is just inhomogeneous generalization of semigroup evolution and it is often called an inhomogeneous semigroup^7^. Note, that contrary to the homogeneous scenario where the time-local generator ${\mathcal {L}}_t = [\partial _t \Lambda _t] \circ \Lambda _t^{-1}$ is defined only for $t \ge 0$ the time dependent generator ${\mathcal {L}}_t$ in (1.5) is defined now for all $t\in {\mathbb {R}}$.

In this paper we propose a particular representation of dynamical maps $\{\Lambda _{t,t_0}\}_{t\ge t_0}$ which by construction satisfy (1.4). Hence, it may be also considered as a particular construction of a legitimate class of memory kernels ${\mathcal {K}}_{t,\tau }$ giving rise to CPTP dynamical maps. Clearly, it is not the most general construction. However, the proposed representation possesses a natural physical interpretation in terms of quantum jumps. Time-local (time convolution-less) approach is discussed as well. It turns out that a time dependent generator also depends upon the initial time $t_0$, i.e. one has a two-parameter family of generators $\{{\mathcal {L}}_{t,t_0}\}_{t\ge t_0}$. Finally, the comparative analysis of traditional time homogeneous versus time inhomogeneous scenario is provided.

## Time homogeneous evolution

### Markovian semigroup

Consider a Markovian semigroup governed by the time independent master equation

2.1 $$\begin{aligned} \partial _t \Lambda _{t,t_0} = {\mathcal {L}} \circ \Lambda _{t,t_0} \ , \ \ \ \Lambda _{t_0,t_0} = \textrm{id} , \end{aligned}$$

where ${\mathcal {L}}$ stands for the GKLS generator (1.2), and $t_0$ is an arbitrary initial time. It is clear that since ${\mathcal {L}}$ does not depend on time the dynamical map depends upon the difference $t-t_0$, i.e. the solution of (2.1) defines one-parameter semigroup $\Lambda _{t,t_0} = \Lambda _{t-t_0}= e^{(t-t_0){\mathcal {L}}}$. Usually, one assumes $t_0=0$ and simply writes $\Lambda _t$. Observe, that any GKLS generator (1.2) can be represented as follows

2.2 $$\begin{aligned} {\mathcal {L}} = \Phi - {\mathcal {Z}} , \end{aligned}$$

where $\Phi , {\mathcal {Z}} : {\mathcal {B}}({\mathcal {H}}) \rightarrow {\mathcal {B}}({\mathcal {H}})$ are linear maps defined by

2.3 $$\begin{aligned} \Phi (\rho ) = \sum _k \gamma _k L_k \rho L_k^\dagger , \ \ \ {\mathcal {Z}}(\rho ) = C\rho + \rho C^\dagger , \end{aligned}$$

with $$C = iH + \frac{1}{2} \sum _k L_k^\dagger L_k$$.

#### Proposition 1

The solution of Eq. (2.1) can be represented via the following series

2.4 $$\begin{aligned} \Lambda _t = \Lambda ^{(0)}_t + \Lambda ^{(0)}_t *\Phi \circ \Lambda ^{(0)}_t + \Lambda ^{(0)}_t *\Phi \circ \Lambda ^{(0)}_t *\Phi \circ \Lambda ^{(0)}_t + \cdots , \end{aligned}$$

where $$\Lambda ^{(0)}_t = e^{- {\mathcal {Z}} t}$$.

#### Proof

let us introduce a *perturbation parameter* $\lambda$ and a one-parameter family of generators

2.5 $$\begin{aligned} {\mathcal {L}}^{(\lambda )} := \lambda \Phi - {\mathcal {Z}} , \end{aligned}$$

such that ${\mathcal {L}} = {\mathcal {L}}^{(\lambda =1)}$. We find a solution to

2.6 $$\begin{aligned} \partial _t \Lambda _{t} = {\mathcal {L}}^{(\lambda )} \circ \Lambda _{t} , \ \ \ \Lambda _{t=0} = \textrm{id} , \end{aligned}$$

as a perturbation series

2.7 $$\begin{aligned} \Lambda _{t} = \Lambda ^{(0)}_{t} + \lambda \Lambda ^{(1)}_{t} + \lambda ^2 \Lambda ^{(2)}_{t} + \cdots. \end{aligned}$$

Inserting the series (2.7) into (2.6) one finds the following infinite hierarchy of equations

2.8 $$\begin{aligned} \partial _t \Lambda ^{(0)}_t= & {} - {\mathcal {Z}} \circ \Lambda ^{(0)}_t , \nonumber \\ \partial _t \Lambda ^{(1)}_t= & {} - {\mathcal {Z}} \circ \Lambda ^{(1)}_t + \Phi \circ \Lambda ^{(0)}_t , \nonumber \\&\vdots&\nonumber \\ \partial _t \Lambda ^{(\ell )}_t= & {} - {\mathcal {Z}} \circ \Lambda ^{(\ell )}_t + \Phi \circ \Lambda ^{(\ell -1)}_t , \nonumber \\&\vdots&\end{aligned}$$

with initial conditions

2.9 $$\begin{aligned} \Lambda ^{(0)}_{t=0} = \textrm{id} \ , \ \ \Lambda ^{(\ell )}_{t=0} = 0 \ , \ (\ell \ge 1). \end{aligned}$$

It is clear that $\Lambda ^{(0)}_t = e^{- {\mathcal {Z}}t}$, and

2.10 $$\begin{aligned} \Lambda ^{(\ell +1)}_{t} = \Lambda ^{(0)}_{t} *\Phi \circ \Lambda ^{(\ell )}_{t} = \Lambda ^{(0)}_{t} *\underbrace{\Phi \circ \Lambda ^{(0)}_{t} *\ldots *\Phi \circ \Lambda ^{(0)}_{t}}_{\ell \ \text{ terms }}. \end{aligned}$$

Finally, fixing $\lambda =1$ the series (2.7) reduces to (2.4). $\square$

Note, that (2.4) is indeed time homogeneous. One finds

2.11 $$\begin{aligned} \Lambda _{t-t_0} = \Lambda ^{(0)}_{t- t_0} + \Lambda ^{(0)}_{t-t_0} *\Phi \circ \Lambda ^{(0)}_{t-t_0} + \Lambda ^{(0)}_{t- t_0} *\Phi \circ \Lambda ^{(0)}_{t- t_0} *\Phi \circ \Lambda ^{(0)}_{t- t_0} + \cdots , \end{aligned}$$

and

2.12 $$\begin{aligned} A_{t-t_0} *B_{t - t_0} := \int _{t_0}^t A_{t-\tau } \circ B_{\tau -t_0}d \tau = \int _{0}^{t-t_0} A_{t-\tau } \circ B_{\tau }d \tau , \end{aligned}$$

does depend upon ‘$t-t_0$’. A series (2.4) is an alternative representation for the conventional exponential representation

2.13 $$\begin{aligned} \Lambda _t = \textrm{id} + {\mathcal {L}} t + \frac{t^2}{2} {\mathcal {L}}^2 + \frac{t^3}{3!} {\mathcal {L}}^3 + \cdots = \textrm{id} + t(\Phi -{\mathcal {Z}}) + \frac{t^2}{2} (\Phi -{\mathcal {Z}})^2 + \frac{t^3}{3!} (\Phi -{\mathcal {Z}})^3 + \cdots. \end{aligned}$$

Note, that contrary to (2.13) each term in (2.4) is completely positive and has a clear physical interpretation: an $\ell$th term reads

2.14 $$\begin{aligned} \Lambda ^{(0)}_{t} *\underbrace{\Phi \circ \Lambda ^{(0)}_{t} *\ldots *\Phi \circ \Lambda ^{(0)}_{t}}_{\ell \ \text{ terms }} = \int _0^t dt_\ell \, \Lambda ^{(0)}_{t-t_\ell } \circ \Phi \circ \int _0^{t_{\ell }} dt_{\ell -1}\,\Lambda ^{(0)}_{t_{\ell } -t_{\ell -1}} \circ \Phi \ldots \circ \Phi \circ \int _0^{t_2} dt_1\, \Lambda ^{(0)}_{t_2-t_1} \circ \Phi \circ \Lambda ^{(0)}_{t_1} , \end{aligned}$$

and it can be interpreted as follows: there are $\ell$ quantum jumps up to time ‘*t*’ at $\{t_1 \le t_2 \le \ldots \le t_\ell \}$ represented by a completely positive map $\Phi$. Between jumps the system evolves according to (unperturbed) completely positive maps $\Lambda ^{(0)}_{t_2-t_1},\Lambda ^{(0)}_{t_3-t_2}, \ldots , \Lambda ^{(0)}_{t_\ell -t_{\ell -1}}$. The series (2.4) represents all possible scenario of $\ell$ jumps for $\ell =0,1,2,\ldots$. By construction, the resulting completely positive map $\Lambda _t$ is also trace-preserving. One often calls (2.4) a *quantum jump* representation of a dynamical map^37–39^. Note, however, that truncating (2.4) at any finite $\ell$ violates trace-preservation since processes with more than $\ell$ jumps are not included. The standard exponential representation (2.13) does not have any clear interpretation. Each separate term $t^k {\mathcal {L}}^k$ does annihilate the trace but is not completely positive. Only the infinite sum of such terms gives rise to completely positive (and trace-preserving) map.

#### Corollary 1

Introducing two completely positive maps $Q_t := \Phi \circ \Lambda ^{(0)}_{t}$ and $\,{\mathcal {P}}_t := \Lambda ^{(0)}_{t} \circ \Phi$ a series (2.4) can be rewritten as follows

2.15 $$\begin{aligned} \Lambda _t= & {} \Lambda ^{(0)}_{t} + \Lambda ^{(0)}_{t} *\Big ( Q_t + Q_t *Q_t + Q_t *Q_t *Q_t + \cdots \Big ) \nonumber \\= & {} \Lambda ^{(0)}_{t} + \Big ( {\mathcal {P}}_t + {\mathcal {P}}_t *{\mathcal {P}}_t + {\mathcal {P}}_t *{\mathcal {P}}_t *{\mathcal {P}}_t + \cdots \Big ) *\Lambda ^{(0)}_{t}. \end{aligned}$$

To summarise: the Markovian semigroup represented in (2.4) is constructed out of the *unperturbed* completely positive and trace non-increasing map $\Lambda ^{(0)}_t= e^{- {\mathcal {Z}} t}$ and the jump operator represented by a completely positive map $\Phi$. These two objects are constrained to satisfy $\textrm{Tr}{\mathcal {L}}(\rho ) = 0$, where ${\mathcal {L}} = \Phi - {\mathcal {Z}}$ defines a GKLS generator.

### Beyond a semigroup

How to generalize (2.4) beyond a semigroup such that time homogeneity is preserved? Suppose that $\Lambda ^{(0)}_t$ is an arbitrary completely positive and trace non-increasing map satisfying $\Lambda ^{(0)}_{t=0} = \textrm{id}$. Let $\{{\mathcal {Z}}_t\}_{t\ge 0}$ be a family of maps such that

2.16 $$\begin{aligned} \partial _t \Lambda ^{(0)}_t = - {\mathcal {Z}}_t *\Lambda ^{(0)}_t , \end{aligned}$$

that is, ${\mathcal {Z}}_t$ is a time non-nonlocal generator of $\Lambda ^{(0)}_t$. Note, that $\Lambda ^{(0)}_t$ defines a semigroup if and only if ${\mathcal {Z}}_t = \delta (t) {\mathcal {Z}}$. Consider a family of jump operators represented by completely positive maps $\{\Phi _t\}_{t \ge 0}$. Define now the following generalization of (2.4)

2.17 $$\begin{aligned} \Lambda _t = \Lambda ^{(0)}_t + \Lambda ^{(0)}_t *\Phi _t *\Lambda ^{(0)}_t + \Lambda ^{(0)}_t *\Phi _t *\Lambda ^{(0)}_t *\Phi _t *\Lambda ^{(0)}_t + \cdots , \end{aligned}$$

that is, one replaces $\Phi \circ \Lambda ^{(0)}_t$ by the convolution $\Phi _t *\Lambda ^{(0)}_t$. By construction (2.17) represents a completely positive map being an infinite sum of completely positive maps

2.18 $$\begin{aligned} \Lambda ^{(\ell )}_t = \Lambda ^{(0)}_{t} *\underbrace{\Phi _t *\Lambda ^{(0)}_{t} *\ldots *\Phi _t *\Lambda ^{(0)}_{t}}_{\ell \ \text{ terms }} , \ \ \ \ell = 1,2,\ldots. \end{aligned}$$

Also a similar quantum jump interpretation still remains true. One finds

2.19 $$\begin{aligned} \Lambda ^{(\ell )}_t = \int _0^t dt_\ell \Lambda ^{(0)}_{t-t_\ell } \circ \ldots \circ \int _0^{t_3} dt_2\, \Phi _{t_3-t_2} \circ \int _0^{t_2} dt_1\, \Lambda ^{(0)}_{t_2-t_1} \circ \int _0^{t_1} d\tau \, \Phi _{t_1-\tau } \circ \Lambda ^{(0)}_\tau. \end{aligned}$$

Between jumps the system evolves according to (unperturbed) completely positive maps $\Lambda ^{(0)}_{t_2-t_1},\Lambda ^{(0)}_{t_3-t_2}, \ldots , \Lambda ^{(0)}_{t_\ell -t_{\ell -1}}$ which are no longer semigroups.

#### Proposition 2

The map represented by (2.17) satisfies the following memory kernel master equation

2.20 $$\begin{aligned} \partial _t \Lambda _t = {\mathcal {K}}_t *\Lambda _t , \ \ \ \Lambda _{t=0} = \textrm{id} , \end{aligned}$$

where

2.21 $$\begin{aligned} {\mathcal {K}}_t = \Phi _t - {\mathcal {Z}}_t. \end{aligned}$$

The map $\Lambda _t$ is trace-preserving if and only if ${\mathcal {K}}_t$ is trace annihilating.

#### Proof

the proof goes the same lines as that of Proposition 1. Introducing

2.22 $$\begin{aligned} {\mathcal {K}}^{(\lambda )}_t = \lambda \Phi _t - {\mathcal {Z}}_t , \end{aligned}$$

and inserting (2.7) into

2.23 $$\begin{aligned} \partial _t \Lambda _t = {\mathcal {K}}^{(\lambda )}_t *\Lambda _t , \ \ \ \Lambda _{t=0} = \textrm{id} , \end{aligned}$$

one obtains the following infinite hierarchy of equations

2.24 $$\begin{aligned} \partial _t \Lambda ^{(0)}_t= & {} - {\mathcal {Z}}_t *\Lambda ^{(0)}_t , \nonumber \\ \partial _t \Lambda ^{(1)}_t= & {} - {\mathcal {Z}}_t *\Lambda ^{(1)}_t + \Phi _t *\Lambda ^{(0)}_t , \nonumber \\&\vdots&\nonumber \\ \partial _t \Lambda ^{(\ell )}_t= & {} - {\mathcal {Z}}_t *\Lambda ^{(\ell )}_t + \Phi _t *\Lambda ^{(\ell -1)}_t , \nonumber \\&\vdots&\end{aligned}$$

with initial conditions (2.9). We show that $\Lambda ^{(\ell )}_{t} = \Lambda ^{(0)}_t *\Lambda ^{(\ell -1)}_{t}$ is a solution to (2.24) which immediately implies (2.18). Indeed, one has

2.25 $$\begin{aligned} \partial _t \Lambda ^{(\ell )}_t = \partial _t [\Lambda ^{(0)}_t *\Phi _t *\Lambda ^{(\ell -1)}_{t}] = \Lambda ^{(0)}_{t=0} \circ [\Phi _t *\Lambda ^{(\ell -1)}_{t}] + [\partial _t \Lambda ^{(0)}_t] *\Phi _t *\Lambda ^{(\ell -1)}_{t} , \end{aligned}$$

and hence using $\partial _t \Lambda ^{(0)}_t = - {\mathcal {Z}}_t *\Lambda ^{(0)}_t$, one obtains

2.26 $$\begin{aligned} \partial _t \Lambda ^{(\ell )}_t = \Phi _t *\Lambda ^{(\ell -1)}_{t} - {\mathcal {Z}}_t *\Lambda ^{(0)}_t *\Phi _t *\Lambda ^{(\ell -1)}_{t} = \Phi _t *\Lambda ^{(\ell -1)}_{t} - {\mathcal {Z}}_t *\Lambda ^{(\ell )}_{t} , \end{aligned}$$

which proves the claim. $\square$

#### Remark 1

Usually on solves the time homogeneous differential equations using the technique of Laplace transform. We provide the alternative proof of Proposition 2 in the Supplementary Information. Here, we provided the proof which can be easily generalized to inhomogeneous case where the Laplace transform technique can not be directly applied.

#### Remark 2

It is clear that if $\Lambda ^{(0)}_t = e^{- {\mathcal {Z}}t}$ is a semigroup, i.e. ${\mathcal {Z}}_t = \delta (t) {\mathcal {Z}}$, then $\Phi _t = \delta (t) \Phi$, and hence

2.27 $$\begin{aligned} {\mathcal {K}}_t = \delta (t)(\Phi - {\mathcal {Z}}) = \delta (t) \, {\mathcal {L}}. \end{aligned}$$

#### Corollary 2

Introducing two completely positive maps $Q_t := \Phi _t *\Lambda ^{(0)}_{t}$ and $\,{\mathcal {P}}_t := \Lambda ^{(0)}_{t} *\Phi _t$ a series (2.17) can be rewritten as follows

2.28 $$\begin{aligned} \Lambda _t = \Lambda ^{(0)}_{t} + \Lambda ^{(0)}_{t} *\Big ( Q_t + Q_t *Q_t + Q_t *Q_t *Q_t + \cdots \Big ) , \end{aligned}$$

or, equivalently,

2.29 $$\begin{aligned} \Lambda _t = \Lambda ^{(0)}_{t} + \Big ( {\mathcal {P}}_t + {\mathcal {P}}_t *{\mathcal {P}}_t + {\mathcal {P}}_t *{\mathcal {P}}_t *{\mathcal {P}}_t + \cdots \Big ) *\Lambda ^{(0)}_{t} , \end{aligned}$$

that is, one has exactly the same representation as in the case of semigroup (2.15). The only difference is the definition of $Q_t$ and ${\mathcal {P}}_t$ in terms of $\Phi _t$ and $\Lambda ^{(0)}_{t}$. Note, however, that if $\Phi _t = \delta (t)\Phi$, then $\Phi _t *\Lambda ^{(0)}_{t} = \Phi \circ \Lambda ^{(0)}_{t}$, i.e. one recovers the same relation as in Corollary 1.

#### Remark 3

It should be stressed that even when $\Phi _t$ is not completely positive, but $Q_t = \Phi _t *\Lambda ^{(0)}_{t}$ is completely positive, then (2.28) is completely positive. Similarly, when $\,{\mathcal {P}}_t := \Lambda ^{(0)}_{t} *\Phi _t$ is completely positive, then (2.29) is completely positive. Hence, complete positivity of $\Phi _t$ is sufficient but not necessary for complete positivity of the dynamical map $\Lambda _t$. Note, however, if $\Phi _t$ is not completely positive the intuitive interpretation of the series (2.17) in terms of quantum jumps is no longer valid.

## Time inhomogeneous evolution

### Time inhomogeneous semigroup

Consider now the dynamical map $\{\Lambda _{t,t_0}\}_{t\ge t_0}$ governed by the time dependent master equation

3.1 $$\begin{aligned} \partial _t \Lambda _{t,t_0} = {\mathcal {L}}_t \circ \Lambda _{t,t_0} \ , \ \ \ \Lambda _{t_0,t_0} = \textrm{id} , \end{aligned}$$

where ${\mathcal {L}}_t$ stands for the time dependent GKLS generator, and $t_0$ is an arbitrary initial time. The corresponding solution has the well known structure

3.2 $$\begin{aligned} \Lambda _{t,t_0} = {\mathcal {T}} \exp \left( \int _{t_0}^t {\mathcal {L}}_\tau d \tau \right) , \end{aligned}$$

where ${\mathcal {T}}$ stands for chronological time ordering. The two-parameter family of maps $\{\Lambda _{t,t_0}\}_{t \ge t_0}$ satisfies the following composition law

3.3 $$\begin{aligned} \Lambda _{t_3,t_2} \circ \Lambda _{t_2,t_1} = \Lambda _{t_3,t_1} , \end{aligned}$$

for any triple $\{t_1, t_2, t_3\}$. Such evolution is evidently CP-divisible. This very property is a generalization of the standard (homogeneous) semigroup property

3.4 $$\begin{aligned} \Lambda _{t_3-t_2} \circ \Lambda _{t_2-t_1} = \Lambda _{t_3-t_1} , \end{aligned}$$

and hence one often calls such maps an inhomogeneous semigroup.

Let us represent the time dependent generator as follows

3.5 $$\begin{aligned} {\mathcal {L}}_t = \Phi _t - {\mathcal {Z}}_t , \end{aligned}$$

where now

3.6 $$\begin{aligned} \Phi _t(\rho ) = \sum _k \gamma _k(t) L_k(t) \rho L_k^\dagger (t) , \ \ \ {\mathcal {Z}}_t(\rho ) = C(t)\rho + \rho C^\dagger (t) , \end{aligned}$$

with $C(t) = iH(t) + \frac{1}{2} \sum _k \gamma _k(t)L_k^\dagger (t) L_k(t)$. To find the corresponding jump representation of $\Lambda _{t,t_0}$ let us introduce the following (inhomogeneous) generalization of the convolution.

#### Definition 1

For any two families of maps $A_{t,t_0}$ and $B_{t,t_0}$

3.7 $$\begin{aligned} (A \circledast B)_{t,t_0} \equiv A_{t,t_0} \circledast B_{t,t_0} := \int _{t_0}^t A_{t,\tau } \circ B_{\tau ,t_0}\, d\tau. \end{aligned}$$

Note, that when $A_{t,t_0}=A_{t-t_0}$ and $B_{t,t_0}=B_{t-t_0}$, then

3.8 $$\begin{aligned} (A \circledast B)_{t,t_0} = \int _{t_0}^t A_{t-\tau } \circ B_{\tau -t_0}\, d\tau = \int _{0}^{t-t_0} A_{t-u} \circ B_{u}\, du = (A *B)_{t-t_0}. \end{aligned}$$

#### Proposition 3

The convolution (3.7) is associative

3.9 $$\begin{aligned} ([A \circledast B] \circledast C)_{t,t_0} = (A \circledast [B \circledast C])_{t,t_0} , \end{aligned}$$

for any three families $A_{t,t_0},\, B_{t,t_0}$ and $C_{t,t_0}$.

See Supplementary Information for the proof.

#### Proposition 4

The solution to (3.1) can be represented via the following series

3.10 $$\begin{aligned} \Lambda _{t,t_0} = \Lambda ^{(0)}_{t,t_0} + \Lambda ^{(0)}_{t,t_0} \circledast (\Phi _t \circ \Lambda ^{(0)}_{t,t_0}) + \Lambda ^{(0)}_{t,t_0} \circledast (\Phi _t \circ \Lambda ^{(0)}_{t,t_0}) \circledast (\Phi _t \circ \Lambda ^{(0)}_{t,t_0}) + \cdots , \end{aligned}$$

where $$\Lambda ^{(0)}_{t,t_0} = {\mathcal {T}} \exp \left( - \int _{t_0}^t {\mathcal {Z}}_\tau d \tau \right)$$.

#### Proof

the proof is a generalization of the proof of Proposition 1. Consider the family of generators

3.11 $$\begin{aligned} {\mathcal {L}}^{(\lambda )}_t := \lambda \Phi _t - {\mathcal {Z}}_t. \end{aligned}$$

We find a solution to

3.12 $$\begin{aligned} \partial _t \Lambda _{t,t_0} = {\mathcal {L}}^{(\lambda )}_t \circ \Lambda _{t,t_0} , \ \ \ \Lambda _{t_0,t_0} = \textrm{id} , \end{aligned}$$

as a perturbation series

3.13 $$\begin{aligned} \Lambda _{t,t_0} = \Lambda ^{(0)}_{t,t_0} + \lambda \Lambda ^{(1)}_{t,t_0} + \lambda ^2 \Lambda ^{(2)}_{t,t_0} + \cdots. \end{aligned}$$

Inserting the series (3.13) into (3.12) one finds the following hierarchy of dynamical equations:

3.14 $$\begin{aligned} \partial _t \Lambda ^{(0)}_{t,t_0}= & {} - {\mathcal {Z}}_t \circ \Lambda ^{(0)}_{t,t_0} , \nonumber \\ \partial _t \Lambda ^{(1)}_{t,t_0}= & {} - {\mathcal {Z}}_t \circ \Lambda ^{(1)}_{t,t_0} + \Phi _t \circ \Lambda ^{(0)}_{t,t_0} , \nonumber \\&\vdots&\nonumber \\ \partial _t \Lambda ^{(\ell )}_{t,t_0}= & {} - {\mathcal {Z}}_t \circ \Lambda ^{(\ell )}_{t,t_0} + \Phi _t \circ \Lambda ^{(\ell -1)}_{t,t_0} , \nonumber \\&\vdots&\end{aligned}$$

with initial conditions

3.15 $$\begin{aligned} \Lambda ^{(0)}_{t_0,t_0} = \textrm{id} , \ \ \ \ \Lambda ^{(\ell )}_{t_0,t_0} = 0 \ \ (\ell > 0). \end{aligned}$$

Clearly, the above hierarchy provides a generalization of (2.8) for the inhomogeneous scenario. Now,

3.16 $$\begin{aligned} \Lambda ^{(0)}_{t,t_0} = {\mathcal {T}} \exp \left( - \int _{t_0}^t {\mathcal {Z}}_\tau d\tau \right) , \end{aligned}$$

defines an inhomogeneous semigroup which is completely positive (but not trace-preserving). As before it is sufficient to show that

3.17 $$\begin{aligned} \Lambda ^{(\ell )}_{t,t_0} = \Lambda ^{(0)}_{t,t_0} \circledast (\Phi _{t} \circ \Lambda ^{(\ell -1)}_{t,t_0}) , \end{aligned}$$

solves (3.14). One finds

3.18 $$\begin{aligned} \partial _t \Lambda ^{(\ell )}_{t,t_0} = \Lambda ^{(0)}_{t,t} \circ \Phi _{t} \circ \Lambda ^{(\ell -1)}_{t,t_0} + [\partial _t\Lambda ^{(0)}_{t,t_0}] \circledast (\Phi _{t} \circ \Lambda ^{(\ell -1)}_{t,t_0}). \end{aligned}$$

Using $\Lambda ^{(0)}_{t,t} = \textrm{id}$, and $\partial _t\Lambda ^{(0)}_{t,t_0} = - {\mathcal {Z}}_t \circ \Lambda ^{(0)}_{t,t_0}$, one gets

3.19 $$\begin{aligned} \partial _t \Lambda ^{(\ell )}_{t,t_0} = \Phi _{t} \circ \Lambda ^{(\ell -1)}_{t,t_0} - [{\mathcal {Z}}_t \circ \Lambda ^{(0)}_{t,t_0}] \circledast (\Phi _{t} \circ \Lambda ^{(\ell -1)}_{t,t_0}) \end{aligned}$$

and finally, observing that

3.20 $$\begin{aligned}{}[{\mathcal {Z}}_t \circ \Lambda ^{(0)}_{t,t_0}] \circledast (\Phi _{t} \circ \Lambda ^{(\ell -1)}_{t,t_0}) = {\mathcal {Z}}_t \circ \Big [ \Lambda ^{(0)}_{t,t_0} \circledast (\Phi _{t} \circ \Lambda ^{(\ell -1)}_{t,t_0})\Big ] = {\mathcal {Z}}_t \circ \Lambda ^{(\ell )}_{t,t_0} , \end{aligned}$$

one completes the proof. $\square$

For an alternative proof which does not use properties of the convolution ‘$\circledast$’ cf. Supplementary Information.

### Beyond an inhomogeneous semigroup

Suppose now that for any initial time $\Lambda ^{(0)}_{t,t_0}$ is an arbitrary completely positive and trace non-increasing map satisfying $\Lambda ^{(0)}_{t_0,t_0} = \textrm{id}$. Let $\{{\mathcal {Z}}_{t,t_0}\}_{t\ge t_0}$ be a family of maps such that

3.21 $$\begin{aligned} \partial _t \Lambda ^{(0)}_{t,t_0} = - {\mathcal {Z}}_{t,t_0} \circledast \Lambda ^{(0)}_{t,t_0} , \end{aligned}$$

that is $\{{\mathcal {Z}}_{t,t_0}\}_{t\ge t_0}$ is a inhomogeneous generalization of $\{{\mathcal {Z}}_t\}_{t\ge 0}$. Now, ${\mathcal {Z}}_{t,t_0}$ does not only depends upon the current time ‘*t*’ but also upon the initial time $t_0$. Define the following generalization of (3.10)

3.22 $$\begin{aligned} \Lambda _{t,t_0} = \Lambda ^{(0)}_{t,t_0} + \Lambda ^{(0)}_{t,t_0} \circledast \Phi _{t,t_0} \circledast \Lambda ^{(0)}_{t,t_0} + \Lambda ^{(0)}_{t,t_0} \circledast \Phi _{t,t_0} \circledast \Lambda ^{(0)}_{t,t_0} \circledast \Phi _{t,t_0} \circledast \Lambda ^{(0)}_{t,t_0} + \cdots , \end{aligned}$$

where $\{\Phi _{t,t_0}\}_{t\ge t_0}$ is a family of completely positive maps which reduces to $\{\Phi _t\}_{t\ge 0}$ in the time homogeneous case. Hence, one replaces $\Phi _t \circ \Lambda ^{(0)}_{t,t_0}$ by the convolution $\Phi _{t,t_0} \circledast \Lambda ^{(0)}_{t,t_0}$. By construction Eq. (3.22) represents a completely positive map being an infinite sum of completely positive maps

3.23 $$\begin{aligned} \Lambda ^{(\ell )}_{t,t_0} = \Lambda ^{(0)}_{t,t_0} \circledast \underbrace{\Phi _{t,t_0} \circledast \Lambda ^{(0)}_{t,t_0} \circledast \ldots \circledast \Phi _{t,t_0} \circledast \Lambda ^{(0)}_{t,t_0} }_{\ell \ \text{ terms }} , \ \ \ \ell = 1,2,\ldots. \end{aligned}$$

Clearly, quantum jump interpretation still remains true.

#### Proposition 5

The map represented by (3.22) satisfies the following memory kernel master equation

3.24 $$\begin{aligned} \partial _t \Lambda _{t,t_0} = {\mathcal {K}}_{t,t_0} \circledast \Lambda _{t,t_0} , \ \ \ \Lambda _{t_0,t_0} = \textrm{id} , \end{aligned}$$

where

3.25 $$\begin{aligned} {\mathcal {K}}_{t,t_0} = \Phi _{t,t_0} - {\mathcal {Z}}_{t,t_0}. \end{aligned}$$

The map $\Lambda _{t,t_0}$ is trace-preserving if and only if ${\mathcal {K}}_{t,t_0}$ is trace annihilating.

#### Proof

the proof goes the same lines as that of Propositions 2 and 4. One easily finds the following hierarchy of equations for maps $\Lambda ^{(\ell )}_{t,t_0}$ defining the series (3.13):

3.26 $$\begin{aligned} \partial _t \Lambda ^{(0)}_{t,t_0}= & {} - {\mathcal {Z}}_{t,t_0} \circledast \Lambda ^{(0)}_{t,t_0} , \nonumber \\ \partial _t \Lambda ^{(1)}_{t,t_0}= & {} - {\mathcal {Z}}_{t,t_0} \circledast \Lambda ^{(1)}_{t,t_0} + \Phi _{t,t_0} \circledast \Lambda ^{(0)}_{t,t_0} , \nonumber \\&\vdots&\nonumber \\ \partial _t \Lambda ^{(\ell )}_{t,t_0}= & {} - {\mathcal {Z}}_{t,t_0} \circledast \Lambda ^{(\ell )}_{t,t_0} + \Phi _{t,t_0} \circledast \Lambda ^{(\ell -1)}_{t,t_0} , \nonumber \\&\vdots&\end{aligned}$$

with initial conditions (3.15). Clearly, the above hierarchy provides a generalization of (2.24) for the inhomogeneous scenario. It is enough to prove that

3.27 $$\begin{aligned} \Lambda ^{(\ell )}_{t,t_0} = \Lambda ^{(0)}_{t,t_0} \circledast \Phi _{t,t_0} \circledast \Lambda ^{(\ell -1)}_{t,t_0}. \end{aligned}$$

One has

3.28 $$\begin{aligned} \partial _t \Lambda ^{(\ell )}_{t,t_0} = \Lambda ^{(0)}_{t,t} \circ \Phi _{t,t_0} \circledast \Lambda ^{(\ell -1)}_{t,t_0} + [\partial _t \Lambda ^{(0)}_{t,t_0}] \circledast \Phi _{t,t_0} \circledast \Lambda ^{(\ell -1)}_{t,t_0}. \end{aligned}$$

Using $\Lambda ^{(0)}_{t,t} = \textrm{id}$, and $\partial _t\Lambda ^{(0)}_{t,t_0} = - {\mathcal {Z}}_{t,t_0} \circledast \Lambda ^{(0)}_{t,t_0}$, one gets

3.29 $$\begin{aligned} \partial _t \Lambda ^{(\ell )}_{t,t_0} = \Phi _{t,t_0} \circledast \Lambda ^{(\ell -1)}_{t,t_0} - {\mathcal {Z}}_{t,t_0} \circledast \Big ( \Lambda ^{(0)}_{t,t_0} \circledast \Phi _{t,t_0} \circledast \Lambda ^{(\ell -1)}_{t,t_0} \Big ) , \end{aligned}$$

and hence

3.30 $$\begin{aligned} \partial _t \Lambda ^{(\ell )}_{t,t_0} = \Phi _{t,t_0} \circledast \Lambda ^{(\ell -1)}_{t,t_0} - {\mathcal {Z}}_{t,t_0} \circledast \partial _t \Lambda ^{(\ell )}_{t,t_0} , \end{aligned}$$

which ends the proof. $\square$

#### Corollary 3

Introducing two completely positive maps $Q_{t,t_0} := \Phi _{t,t_0} \circledast \Lambda ^{(0)}_{t,t_0}$ and $\,{\mathcal {P}}_{t,t_0} := \Lambda ^{(0)}_{{t,t_0}} \circledast \Phi _{t,t_0}$ a series (2.17) can be rewritten as follows

3.31 $$\begin{aligned} \Lambda _{t,t_0} = \Lambda ^{(0)}_{{t,t_0}} + \Lambda ^{(0)}_{{t,t_0}} \circledast \Big ( Q_{t,t_0} + Q_{t,t_0} \circledast Q_{t,t_0} + Q_{t,t_0} \circledast Q_{t,t_0} \circledast Q_{t,t_0} + \cdots \Big ) , \end{aligned}$$

or, equivalently,

3.32 $$\begin{aligned} \Lambda _{t,t_0} = \Lambda ^{(0)}_{{t,t_0}} + \Big ( {\mathcal {P}}_{t,t_0} + {\mathcal {P}}_{t,t_0} \circledast {\mathcal {P}}_{t,t_0} + {\mathcal {P}}_{t,t_0} \circledast {\mathcal {P}}_{t,t_0} \circledast {\mathcal {P}}_{t,t_0} + \cdots \Big ) \circledast \Lambda ^{(0)}_{t,t_0}. \end{aligned}$$

They reduce to (2.28) and (2.29) in the time homogeneous case.

Table 1 summarizes the construction of time homogeneous versus time inhomogeneous dynamical maps.

**Table 1:** Representation of dynamical maps: time homogeneous versus inhomogeneous case

	Time homogeneous	Time inhomogeneous
General map	$\Lambda ^{(\ell )}_{t-t_0} = \Lambda ^{(0)}_{t-t_0} *\Phi _{t-t_0} * \Lambda ^{(\ell -1)}_{t-t_0}$	$\Lambda ^{(\ell )}_{t,t_0} = \Lambda ^{(0)}_{t,t_0} \circledast \Phi _{t,t_0} \circledast \Lambda ^{(\ell -1)}_{t,t_0}$
Markovian semigroup	$\Lambda ^{(\ell )}_{t-t_0} = \Lambda ^{(0)}_{t-t_0} *(\Phi \circ \Lambda ^{(\ell -1)}_{t-t_0})$	$\Lambda ^{(\ell )}_{t,t_0} = \Lambda ^{(0)}_{t,t_0} \circledast (\Phi _{t} \circ \Lambda ^{(\ell -1)}_{t,t_0})$

## Time local approach

Very often describing the evolution of an open system one prefers to use a time-local (or so-called convolutionless (TCL)) approach^1^. Formally, in the time homogeneous case given a dynamical map $\{\Lambda _t\}_{t\ge 0}$ one defines the corresponding time-local generator ${\mathcal {L}}_t := [\partial _t \Lambda _t] \circ \Lambda _t^{-1}$ (assuming that $\Lambda _t$ is invertible). This way the map $\Lambda _t$ satisfies

4.1 $$\begin{aligned} \partial _t \Lambda _t = {\mathcal {L}}_t \circ \Lambda _ t. \end{aligned}$$

This procedure might be a bit confusing since (4.1) coincides with (3.1) for the inhomogeneous map $\Lambda _{t,t_0}$. To clarify this point let us introduce again an initial time and consider $\Lambda _{t,t_0} = \Lambda _{t-t_0}$. Now, the time-local generator reads

4.2 $$\begin{aligned} {\mathcal {L}}_{t-t_0} := [\partial _t \Lambda _{t-t_0}] \circ \Lambda _{t-t_0}^{-1} , \end{aligned}$$

that is, the generator does depend upon the initial time^40^. It implies that the corresponding propagators

4.3 $$\begin{aligned} V_{t,s} := \Lambda _{t-t_0} \circ \Lambda ^{-1}_{s-t_0} = {\mathcal {T}} \exp \left( \int _s^t {\mathcal {L}}_{\tau - t_0} d\tau \right) = {\mathcal {T}} \exp \left( \int _{s-t_0}^{t-t_0} {\mathcal {L}}_{\tau } d\tau \right) , \end{aligned}$$

also does depend upon $t_0$. Clearly, fixing $t_0=0$ this fact is completely hidden. The dependence upon $t_0$ drops out only in the semigroup case when ${\mathcal {L}}_{t-t_0} = {\mathcal {L}}$.

Similar analysis may be applied to inhomogeneous scenario as well. Now, instead of convolution (3.21) one may define a time-local generator

4.4 $$\begin{aligned} {\mathcal {L}}_{t,t_0} := [\partial _t \Lambda _{t,t_0}] \circ \Lambda _{t,t_0}^{-1} , \end{aligned}$$

such that $\Lambda _{t,t_0}$ satisfies the following inhomogeneous TCL master equation

4.5 $$\begin{aligned} \partial _t \Lambda _{t,t_0} = {\mathcal {L}}_{t,t_0} \circ \Lambda _{t,t_0} . \end{aligned}$$

Again, the corresponding propagator

4.6 $$\begin{aligned} V_{t,s} := \Lambda _{t,t_0} \circ \Lambda ^{-1}_{s,t_0} = {\mathcal {T}} \exp \left( \int _s^t {\mathcal {L}}_{\tau ,t_0} d\tau \right) , \end{aligned}$$

also does depend upon $t_0$. Hence, the local composition law

4.7 $$\begin{aligned} V_{t,s} \circ V_{s,u} = V_{t,u} , \end{aligned}$$

holds only if the above propagators are defined w.r.t. the same initial time. Otherwise, composing the propagators does not have any sense. Equation (4.5) reduces to (3.1) only if ${\mathcal {L}}_{t,t_0}$ does not depend upon $t_0$. In this case one recovers an inhomogeneous semigroup and ${\mathcal {L}}_{t,t_0} = {\mathcal {L}}_{t}$.

## Conclusions

We have constructed a family of time inhomogeneous dynamical maps $\{\Lambda _{t,t_0}\}_{t\ge 0}$ represented by the following infinite series

5.1 $$\begin{aligned} \Lambda _{t,t_0} = \Lambda ^{(0)}_{t,t_0} + \Lambda ^{(1)}_{t,t_0} + \Lambda ^{(2)}_{t,t_0} + \cdots , \end{aligned}$$

where each single map $\Lambda ^{(\ell )}_{t,t_0}$ is completely positive. Moreover, the construction does guarantee that $\Lambda _{t,t_0}$ is trace-preserving. Each map $\Lambda ^{(\ell )}_{t,t_0}$ represents a process with $\ell$ quantum jumps occurring in the interval $[t_0,t]$. The ‘free’ evolution (no jumps) corresponds to $\Lambda ^{(0)}_{t,t_0}$. Quantum jumps are represented by a family of completely positive maps $\{\Phi _{t,t_0}\}_{t\ge t_0}$ such that $\Lambda ^{(\ell )}_{t,t_0}$ is represented as in the Table 1.

In the time-homogeneous case the above representation simplifies to

5.2 $$\begin{aligned} \Lambda _{t-t_0} = \Lambda ^{(0)}_{t-t_0} + \Lambda ^{(1)}_{t-t_0} + \Lambda ^{(2)}_{t-t_0} + \cdots , \end{aligned}$$

with a similar interpretation. The dynamical map $\Lambda _{t,t_0}$ satisfies the corresponding Nakajima-Zwanzig memory kernel master equation or equivalently time-local (TCL) master equation displayed in the Table 2.

**Table 2:** Dynamical equations: time homogeneous versus inhomogeneous case

	Time homogeneous	Time inhomogeneous
Memory kernel ME	$\partial _t \Lambda _{t-t_0} = {\mathcal {K}}_{t-t_0} *\Lambda _{t-t_0}$	$\partial _t \Lambda _{t,t_0} = {\mathcal{K}}_{t,t_0}\circledast\Lambda _{t,t_0}$
Markovian semigroup	${\mathcal {K}}_{t-t_0} = \delta (t-t_0)\, {\mathcal {L}}$	${\mathcal {K}}_{t,\tau } = \delta (t-\tau )\, {\mathcal {L}}_t$
TCL ME	$\partial _t \Lambda _{t-t_0} = {\mathcal {L}}_{t-t_0} \circ \Lambda _{t-t_0}$	$\partial _t \Lambda _{t,t_0} = {\mathcal {L}}_{t,t_0} \circ \Lambda _{t,t_0}$
TCL generator	${\mathcal {L}}_{t-t_0} = [\partial _t \Lambda _{t-t_0}] \circ \Lambda _{t-t_0}^{-1}$	${\mathcal {L}}_{t,t_0} = [\partial _t \Lambda _{t,t_0}] \circ \Lambda _{t,t_0}^{-1}$
New ME	$\partial _t \Lambda _{t-t_0} = {\mathbb {K}}_{t-t_0} *\Lambda _{t-t_0}+\partial _t \Lambda _{t-t_0}$	$\partial _t \Lambda _{t,t_0} = {\mathbb {K}}_{t,t_0} \circledast \Lambda _{t,t_0} + \partial _t \Lambda _{t,t_0}^{(0)}$

Interestingly, apart from Nakajima-Zwanzing memory kernel master equation the map $\Lambda _{t,t_0}$ satisfies the following dynamical equation

5.3 $$\begin{aligned} \partial _t \Lambda _{t,t_0} = {\mathbb {K}}_{t,t_0} \circledast \Lambda _{t,t_0} + \partial _t \Lambda _{t,t_0}^{(0)} , \end{aligned}$$

where the new kernel ${\mathbb {K}}_{t,t_0}$ is defined by

5.4 $$\begin{aligned} {\mathbb {K}}_{t,t_0} = \partial _t {\mathcal {P}}_{t,t_0} = \partial _t[ \Phi _{t,t_0} \circledast \Lambda _{t,t_0}^{(0)}] , \end{aligned}$$

that is, it is constructed in terms of the ‘free’ evolution represented by $\Lambda _{t,t_0}^{(0)}$ and the jump operators $\Phi _{t,t_0}$ (the details of the derivation are presented in the Supplementary Information).

This is very general class of legitimate quantum evolutions and corresponding dynamical equations. It would be interesting to apply the above scheme to discuss time inhomogeneous semi-Markov processes^28,29,33,41^ and collision models (cf.^42^ for the recent review).

## Supplementary Information

Supplementary Information.

## Data Availability

All data generated or analysed during this study are included in this published article and its supplementary information file.
