# Supplementary material for: Time inhomogeneous quantum dynamical maps
Source: Sci Rep. 2022 Dec 8;12:21223. doi: 10.1038/s41598-022-25694-1 (PMC9731978; doi:10.1038/s41598-022-25694-1)
Supplement: Supplementary file 1 — Supplementary Information. [file 41598_2022_25694_MOESM1_ESM.pdf]

Supplementary Information for Time inhomogeneous quantum dynamical maps

Dariusz Chruściński
Institute of Physics, Faculty of Physics, Astronomy and Informatics
Nicolaus Copernicus University
Grudziadzka 5/7, 87-100 Toruń, Poland

1 Proof of Proposition 2

The simplest way to solve (2.24) is to pass to the Laplace Transform (LT) domain:

$$\widetilde{F}_s := \int_0^\infty e^{-ts} F_t dt. \tag{1.1}$$

Taking LT of (2.24) one obtains

$$s\widetilde{\Lambda}_s^{(\ell)} - \mathrm{id} = -\widetilde{\mathcal{Z}}_s \circ \widetilde{\Lambda}_s^{(\ell)} + \widetilde{\Phi}_s \circ \widetilde{\Lambda}_s^{(\ell)}, \tag{1.2}$$

and using

$$s\widetilde{\Lambda}_s^{(0)} - \mathrm{id} = -\widetilde{\mathcal{Z}}_s \circ \widetilde{\Lambda}_s^{(0)}, \tag{1.3}$$

one finds

$$s\widetilde{\Lambda}_s^{(\ell)} = (s + \widetilde{Z}_s)^{-1} \circ \widetilde{\Phi}_s \circ \widetilde{\Lambda}_s^{(\ell-1)} = \widetilde{\Lambda}_s^{(0)} \circ \widetilde{\Phi}_s \circ \widetilde{\Lambda}_s^{(\ell-1)}, \tag{1.4}$$

and hence going back to the time domain one finally obtains $\Lambda_t^{(\ell)} = \Lambda_t^{(0)} * \Phi_t * \Lambda_t^{(\ell-1)}$ which implies (3.23).

2 Proof of Proposition 3

One has

$$(A \circledast [B \circledast C])_{t,t_0} = \int_{t_0}^t d\tau\, A_{t,\tau} \circ [B \circledast C]_{\tau,t_0} = \int_{t_0}^t d\tau\, A_{t,\tau} \circ \int_{t_0}^\tau du B_{\tau,u} \circ C_{u,t_0}, \tag{2.1}$$

and hence using

$$\int_{t_0}^t d\tau \int_{t_0}^\tau du \ldots = \int_{t_0}^t du \int_u^t d\tau \ldots, \tag{2.2}$$

one obtains

$$(A \circledast [B \circledast C])_{t,t_0} = \int_{t_0}^t du \left\{ \int_u^t d\tau\, A_{t,\tau} \circ B_{\tau,u} \right\} \circ C_{u,t_0} = \int_{t_0}^t du [A \circledast B]_{t,u} \circ C_{u,t_0} = ([A \circledast B] \circledast C)_{t,t_0}, \tag{2.3}$$

which ends the proof. □

3 Proof of Proposition 4

One finds

$$\Lambda^{(\ell)}_{t,t_0} = \Lambda^{(0)}_{t,t_0} \circ C^{(\ell)}_{t,t_0}, \tag{3.1}$$

where C_{t,t_0} satisfies

$$\partial_t C^{(\ell)}_{t,t_0} = (\Lambda^{(0)}_{t,t_0})^{-1} \circ \Phi_t \circ \Lambda^{(\ell-1)}_{t,t_0} \ , \quad C^{(\ell)}_{t_0,t_0} = 0, \tag{3.2}$$

and hence

$$C^{(\ell)}_{t,t_0} = \int_{t_0}^{t} (\Lambda^{(0)}_{\tau,t_0})^{-1} \circ \Phi_\tau \circ \Lambda^{(\ell-1)}_{\tau,t_0}, \tag{3.3}$$

which eventually gives rise to

$$\Lambda^{(\ell)}_{t,t_0} = \int_{t_0}^{t} \Lambda^{(0)}_{t,\tau} \circ \Phi_\tau \circ \Lambda^{(\ell-1)}_{\tau,t_0} d\tau = \Lambda^{(0)}_{t,t_0} \circledast (\Phi_t \circ \Lambda^{(\ell-1)}_{t,t_0}), \tag{3.4}$$

where we have used the semigroup property

$$\Lambda^{(0)}_{t,t_0} \circ (\Lambda^{(0)}_{\tau,t_0})^{-1} = \Lambda^{(0)}_{t,t_0} \circ \Lambda^{(0)}_{t_0,\tau} = \Lambda^{(0)}_{t,\tau}. \tag{3.5}$$

Simple iteration leads to

$$\Lambda^{(\ell+1)}_{t} = \Lambda^{(0)}_{t} \circledast (\Phi \circ \Lambda^{(\ell)}_{t}) = \Lambda^{(0)}_{t} \circledast \underbrace{(\Phi_t \circ \Lambda^{(0)}_{t}) \circledast \ldots \circledast (\Phi_t \circ \Lambda^{(0)}_{t,t_0})}_{\ell \text{ terms}} . \tag{3.6}$$

which proves (3.10). □

4 Derivation of (5.3)

Defining $\mathcal{P}_{t,t_0} = \Phi_{t,t_0} \circledast \Lambda^{(0)}_{t,t_0}$ one has

$$\Lambda_{t,t_0} = \Lambda^{(0)}_{t,t_0} + \Big(\mathcal{P}_{t,t_0} + \mathcal{P}_{t,t_0} \circledast \mathcal{P}_{t,t_0} + \mathcal{P}_{t,t_0} \circledast \mathcal{P}_{t,t_0} \circledast \mathcal{P}_{t,t_0} + \ldots\Big) \circledast \Lambda^{(0)}_{t,t_0}, \tag{4.1}$$

and hence

$$\partial_t \Lambda_{t,t_0} = \partial_t \Lambda^{(0)}_{t,t_0} + \partial_t \Big(\mathcal{P}_{t,t_0} + \mathcal{P}_{t,t_0} \circledast \mathcal{P}_{t,t_0} + \mathcal{P}_{t,t_0} \circledast \mathcal{P}_{t,t_0} \circledast \mathcal{P}_{t,t_0} + \ldots\Big) \circledast \Lambda^{(0)}_{t,t_0}, \tag{4.2}$$

due to $\mathcal{P}_{t,t} = 0$. Now,

$$\begin{aligned} &\partial_t \Big(\mathcal{P}_{t,t_0} + \mathcal{P}_{t,t_0} \circledast \mathcal{P}_{t,t_0} + \mathcal{P}_{t,t_0} \circledast \mathcal{P}_{t,t_0} \circledast \mathcal{P}_{t,t_0} + \ldots\Big) \circledast \Lambda^{(0)}_{t,t_0} \\ &= \Big(\partial_t \mathcal{P}_{t,t_0} + [\partial_t \mathcal{P}_{t,t_0}] \circledast \mathcal{P}_{t,t_0} + [\partial_t \mathcal{P}_{t,t_0}] \circledast \mathcal{P}_{t,t_0} \circledast \mathcal{P}_{t,t_0} + \ldots\Big) \circledast \Lambda^{(0)}_{t,t_0} \\ &= [\partial_t \mathcal{P}_{t,t_0}] \circledast \Lambda^{(0)}_{t,t_0} + [\partial_t \mathcal{P}_{t,t_0}] \circledast \Big(\mathcal{P}_{t,t_0} + \mathcal{P}_{t,t_0} \circledast \mathcal{P}_{t,t_0} + \mathcal{P}_{t,t_0} \circledast \mathcal{P}_{t,t_0} \circledast \mathcal{P}_{t,t_0} + \ldots\Big) \circledast \Lambda^{(0)}_{t,t_0} \\ &= [\partial_t \mathcal{P}_{t,t_0}] \circledast \Lambda^{(0)}_{t,t_0}, \end{aligned} \tag{4.3}$$

which proves (5.3).
